# Identifying Shared Risk Genes for Asthma, Hay Fever, and Eczema by Multi-Trait and Multiomic Association Analyses

**DOI:** 10.3389/fgene.2020.00270

**Published:** 2020-04-16

**Authors:** Hongping Guo, Jiyuan An, Zuguo Yu

**Affiliations:** ^1^Key Laboratory of Intelligent Computing and Information Processing of Ministry of Education and Hunan Key Laboratory for Computation and Simulation in Science and Engineering, Xiangtan University, Hunan, China; ^2^School of Mathematics and Computer Science, Hanjiang Normal University, Hubei, China; ^3^Centre for Tropical Crops and Biocommodities, Queensland University of Technology, Brisbane, QLD, Australia; ^4^School of Electrical Engineering and Computer Science, Queensland University of Technology, Brisbane, QLD, Australia

**Keywords:** asthma, hay fever, eczema, association studies, shared genes, multi-trait, multiomic

## Abstract

Asthma, hay fever and eczema are three comorbid diseases with high prevalence and heritability. Their common genetic architectures have not been well-elucidated. In this study, we first conducted a linkage disequilibrium score regression analysis to confirm the strong genetic correlations between asthma, hay fever and eczema. We then integrated three distinct association analyses (metaCCA multi-trait association analysis, MAGMA genome-wide and MetaXcan transcriptome-wide gene-based tests) to identify shared risk genes based on the large-scale GWAS results in the GeneATLAS database. MetaCCA can detect pleiotropic genes associated with these three diseases jointly. MAGMA and MetaXcan were performed separately to identify candidate risk genes for each of the three diseases. We finally identified 150 shared risk genes, in which 60 genes are novel. Functional enrichment analysis revealed that the shared risk genes are enriched in inflammatory bowel disease, T cells differentiation and other related biological pathways. Our work may provide help on treatment of asthma, hay fever and eczema in clinical applications.

## Introduction

Asthma is a bronchial disease characterized by chronic inflammation and narrowing of the airways. It results in recurring coughing, periods of wheezing, chest tightness, and mucus production (Moffatt et al., 2010; Vicente et al., 2017; Pividori et al., 2019). Hay fever (allergic rhinitis) is an inflammation disease of the nasal mucous membranes. Its symptoms include sneezing, nasal congestion, rhinorrhea, and itching (Ramasamy et al., 2011; Bunyavanich et al., 2014; Ferreira et al., 2014). Eczema (atopic dermatitis) is a form of dermatitis. Its manifestations include itching and dryness, recurring skin rashes with redness, blistering and skin edema (Sun et al., 2011; Weidinger et al., 2013; Paternoster et al., 2015). The three diseases have high global prevalence. Nearly 15% of the world population are affected by asthma (Vicente et al., 2017), 10∼20% by hay fever (Ober and Yao, 2011), 15∼30% of children and 5∼10% of adults are affected by eczema (Waage et al., 2018). Poor life quality and substantial medical expenditure bother the patients (Ober and Yao, 2011; Waage et al., 2018). Moreover, the three diseases have significant genetic contributions in different patients. The heritability ranges from 35% to 95% for asthma, from 33% to 91% for hay fever and from 71% to 84% for eczema (Ober and Yao, 2011; Zhu et al., 2018; Johansson et al., 2019). Genome-wide association studies (GWAS) are the most powerful tools to identify the disease-associated variants. GWAS have been carried out separately for asthma, hay fever and eczema in the last two decades (Moffatt et al., 2010; Paternoster et al., 2015; Waage et al., 2018). To date (2019.11), hundreds of statistically significant single-nucleotide polymorphisms (SNPs) have been identified to be associated with each of three diseases according to GWAS-catalog database (MacArthur et al., 2017).

Clinical and epidemiological studies have found that the three diseases often co-occur in the same person or different members from the same family (Ober and Yao, 2011; Ferreira et al., 2017). Up to 90% of asthmatics suffer from allergic diseases such as hay fever and eczema (Leynaert et al., 2000; Zhu et al., 2018). Furthermore, eczema was demonstrated to be a major risk factor for the development of asthma and hay fever (Spergel, 2010). About 30% eczema patients were affected by asthma, and approximately 66% eczema patients were affected by hay fever (Ober and Yao, 2011). Similarly, 19∼38% hay fever patients were affected by asthma simultaneously (Ober and Yao, 2011). These phenomena indicate potential genetic pleiotropy and co-morbidity between asthma, hay fever and eczema. Therefore, identifying shared risk genes between these three diseases can broaden our knowledge of the underlying shared genetic causes, as well as lead the way to prevention and treatments based on the molecular mechanisms (Marenholz et al., 2013; Ferreira et al., 2017; Zhu et al., 2018).

In the past 3 years, several large-scale GWAS focused on unraveling the shared genetic architectures between asthma, hay fever and eczema based on data from UK Biobank (Sudlow et al., 2015; Ferreira et al., 2017; Zhu et al., 2018; Johansson et al., 2019). Researchers (Ferreira et al., 2017) performed meta-analysis of allergic diseases (asthma and/or hay fever and/or eczema) based on GWAS results from 13 studies by using METAL (Willer et al., 2010) software to identify the associations, and used GeneNetwork (Fehrmann et al., 2015) to identify biological processes enriched among the genes. Finally the reason why asthma, hay fever and eczema partly coexist was revealed, i.e., they share many genetic variations that dysregulate the expression of immune-related genes. Subsequently, another study (Zhu et al., 2018) applied cross-trait GWAS meta-analysis by using R package ASSET (Bhattacharjee et al., 2012) to combine the associations for asthma and allergic diseases (hay fever and/or eczema) at individual variants. They demonstrated that shared risk loci not only influence immune/inflammatory systems but also tissues with epithelium cells. A recent work showed that these three diseases shared a large amount of genetic contributions, but part of which is more disease specific (Johansson et al., 2019). However, these studies did not make strict distinction between the three diseases in phenotypic definition. Either they used a broad allergic disease defined as asthma and/or hay fever and/or eczema, or a slightly more narrow definition which distinguished asthma from allergic diseases, i.e., asthma and allergic diseases (hay fever and/or eczema). This may cause inaccurate conclusions. Moreover, the pleiotropic effect between each gene (including multiple variants) and these three correlated diseases jointly were not taken into account, which may lead to low statistical power or small percentage of explainable genetic variance. Multi-trait association study method metaCCA (Cichonska et al., 2016) enables the pleiotropy to be resolved effectively. It has been applied to identify shared pleiotropic genes for three correlated diseases (type 2 diabetes, obesity and dyslipidemia) (Chen et al., 2018) and five major psychiatric disorders (Jia et al., 2019), respectively. However, the sample sizes in the above-mentioned two studies were not large enough (several tens of thousands), and only genome data was used, resulting in only 25 and 66 shared risk genes obtained, separately.

In this study, we firstly performed a linkage disequilibrium (LD) score regression to evaluate genetic correlations between asthma, hay fever and eczema. We then integrated three distinct association analyses (metaCCA multi-trait association analysis, MAGMA genome-wide and MetaXcan transcriptome-wide gene-based tests) to identify shared risk genes based on the large-scale GWAS results in GeneATLAS database (Canela-Xandri et al., 2018). MetaCCA can detect pleiotropic genes jointly associated with these three diseases (Cichonska et al., 2016). MAGMA (de Leeuw et al., 2015) considers the correlations between genes and each disease, and MetaXcan (Gamazon et al., 2015) merges the gene expression information to identify candidate risk genes for each of the three diseases. Through these three different analyses, we obtained the potential shared risk genes associated with these three diseases. Finally we verified them by GWAS-catalog analysis, enrichment analysis and protein–protein interaction (PPI) network analysis to provide biology insights.

## Materials and Methods

### GWAS Result Datasets

We downloaded the GWAS results from a publicly accessible database GeneATLAS (Canela-Xandri et al., 2018), including asthma (*N*_*cases*_ = 52269, *N*_*controls*_ = 399995), hay fever (*N*_*cases*_ = 25473, *N*_*controls*_ = 426791) and eczema (*N*_*cases*_ = 11552, *N*_*controls*_ = 440712). The total 452264 samples are all European-ancestry individuals from UK Biobank. In this study, we used the same 623944 genotyped variants in each sample that passed quality control in GeneATLAS.

### Methods

#### LD Score Regression Analysis

We applied linkage disequilibrium score regression (LDSC) (Bulik-Sullivan et al., 2015) to estimate genetic correlations, as well as SNP heritability and LD-score intercept for asthma, hay fever and eczema, respectively. We used the reference panel from European-ancestry population of 1000 Genome Project Phase 3 (The 1000 Genomes Project Consortium, 2015).

#### Multi-Trait Association Analysis

After estimating genetic correlations between asthma, hay fever and eczema, we used metaCCA multi-trait GWAS approach to identify pleiotropic genes associated equally with the three diseases. MetaCCA enables the measure of correlation between the gene (including multiple variants) and multiple traits using canonical correlation analysis (CCA) (Cichonska et al., 2016). This takes into consideration that there exist dependencies (i.e., covariances) between genotypic and phenotypic variables, and the cross-covariance between all genotypic and phenotypic variables is made of univariate regression coefficients in linear model.

In order to reduce the computation time and memory, we first conducted gene annotation by referring NCBI human genome build 37 (including 19427 gene locations), and found that 301949 (48.39%) of the total 623944 SNPs are mapped to 17446 genes. Then we performed linkage disequilibrium (LD) based pruning to filter SNPs using PLINK software (version: 1.90b) with parameters (–indep-pairwise 50 5 0.2) (Jia et al., 2019), i.e., calculating LD between each pair of SNPs in a window of 50 SNPs, removing one of a pair of SNPs if the LD is greater than 0.2, shifting the window of 5 SNPs forward and repeating the procedure until no pairs of SNPs with high LD remain. We selected those SNPs which overlap with variants from the European population in HapMap3. After pruning, 24946 of the input 301949 SNPs are mapped to 6575 genes. We used 24946 SNPs to estimate genotypic correlation structure. 301949 SNPs were applied to estimate phenotypic correlation structure due to the fact that the larger number of variants, the higher the estimation accuracy (Cichonska et al., 2016). The covariance matrix between all genotypic and phenotypic variables is made up of regression coefficients in the GWAS results. The majority of the CPU memory in metaCCA is spent on estimating the covariance between genotypic variables. The space complexity is O(n^2^), where n is the number of SNPs, and it used about 6.3 gb memory for 24946 SNPs. MetaCCA mainly uses CPU time in estimation of genotypic correlation structure and canonical correlations. In our study, metaCCA took about 4 h for multi-trait gene test of the three diseases. We performed the operations on a computer of Intel Xeon E5-2640 CPU 2.40 GHz.

To determine significant loci (*p* < 5 × 10^–8^) that are independent from each other, we used the clump procedure of PLINK software (Purcell et al., 2007). We set parameters (–clump-p1 5 × 10^–8^ –clump-p2 1 × 10^–5^ –clump-r2 0.2 –clump-kb 500) (Zhu et al., 2018) indicating the SNPs with a *p*-value less than 1 × 10^–5^, LD statistic *r*^2^ more than 0.2, and within 500 kb distance from the peak, will be assigned to that peak’s clump.

#### Genome-Wide Gene-Based Analysis

Gene-based analysis is a statistical method for simultaneous analysis of multiple genetic variations to determine their joint effect. MAGMA, a genome-wide gene-based association method based on a multiple linear principal components regression model (de Leeuw et al., 2015), was used to identify significant genes using the GWAS results for asthma, hay fever and eczema, respectively. We regarded the individual-level genotype data from European-ancestry population of 1000 Genomes Project Phase 3 as reference. 19427 genes in the whole genome were used to determine the significance threshold in Bonferroni correction. The space complexity of MAGMA is O(k^2^), where k is the number of genes. For a human genome, the required memory is about 5 gb. In MAGMA, the majority of the CPU time is spent on the ordinary least squares method, the time complexity is O(k^2^ × (n + k)), where k is the number of genes and n is the number of SNPs. In our study, MAGMA took about 1 min to analyze each disease.

#### Transcriptome-Wide Gene-Based Analysis

We used the MetaXcan framework to integrate expression quantitative trait loci (eQTL) information with GWAS results and map genes associated with disease traits. MetaXcan is a transcriptome-wide gene-based association approach that estimates tissue-specific gene expression profiles from GWAS results using prediction models trained in large reference databases, and correlates predicted expression levels with diseases (such as asthma) to detect potential disease-associated genes (Barbeira et al., 2018). It has high concordance (correlation coefficient: *R*^2^ > 0.999) with the individual-level version PrediXcan (Gamazon et al., 2015). Training sets are reference transcriptome datasets from the Genotype-Tissue Expression Project (GTEx: version 7) (GTEx Consortium, 2017), the weights and covariances of prediction model for different tissues are available from PredictDB (http://predictdb.org/).

In order to reduce multiple-testing burden, we analyzed 10 of the total 48 tissues, 4 obvious tissues (Whole Blood, Lung, Skin Sun Exposed and Skin Not Sun Exposed) plus 6 other relevant tissues (Cells EBV-transformed lymphocytes, Cells Transformed fibroblasts, Esophagus Gastroesophageal Junction, Esophagus Mucosa, Esophagus Muscularis and Vagina) reported in previous studies (Ferreira et al., 2017; Zhu et al., 2018). The total number of genes (27314) in the 10 tissues was used to determine the Bonferroni correction threshold. We ran MetaXcan separately in asthma, hay fever and eczema, each with the same 10 tissues, and used per SNP *p*-value from GWAS results after correction for the LD-score intercept. MetaXcan uses a small amount of memory and very little CPU time. MetaXcan’s CPU time is primarily spent on the calculation of covariance of the gene matrix. The space and time complexity are O(*k*^2^) and O(*k*^3^) respectively, where *k* is the number of genes in the tissue. In our study, 18 min were spent on MetaXcan’s analysis of 10 tissues for each disease.

#### GWAS-Catalog Analysis, Enrichment Analysis and PPI Network Analysis

To understand whether the identified genes have been reported in the previous GWAS studies for asthma, hay fever and eczema, we downloaded the corresponding GWAS catalog from NHGRI-EBM (3 November, 2019), and searched the genes one by one. To gain biology insights from the shared risk genes, we performed KEGG (Kyoto Encyclopedia of Genes and Genomes) pathway analysis using the Enrichr web server (Kuleshov et al., 2016) from http://amp.pharm.mssm.edu/Enrichr. The significant criterion is that the adjusted *p*-value is less than 0.05. In addition, we used STRING v10 (Szklarczyk et al., 2015) from https://string-db.org/ to analyze the PPI network.

**FIGURE 1:**
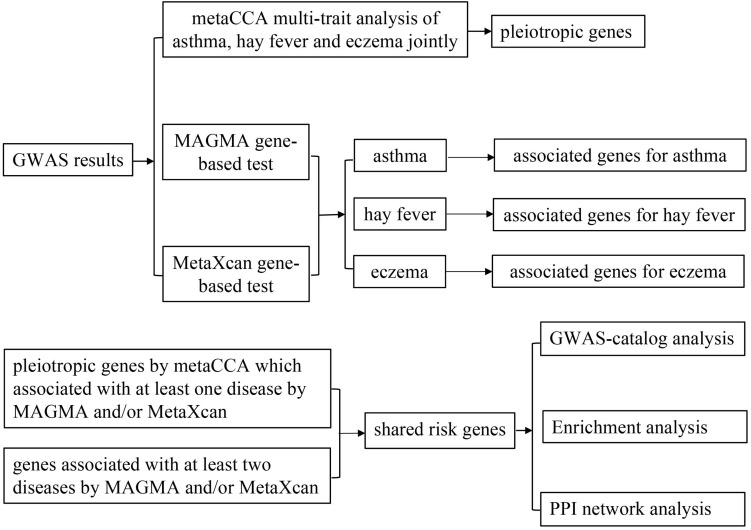
Flow chart of the present work.

A flow chart of our work is shown in Figure 1. That is, we integrated three association studies (metaCCA multi-trait association analysis, MAGMA genome-wide and MetaXcan transcriptome-wide gene-based tests) to identify candidate risk genes, and then conducted GWAS-catalog analysis, enrichment analysis and PPI network analysis to the shared risk genes.

## Results

### Genetic Correlation Between Asthma, Hay Fever and Eczema

We evaluated the genetic correlation between asthma, hay fever and eczema using LD score regression (LDSC). Genetic correlation between asthma and hay fever (*r*_*g*_ = 0.665, *SE* = 0.0457, *P* = 5.26 × 10^–48^) is the strongest, followed by the correlation between asthma and eczema (*r*_*g*_ = 0.4519, *SE* = 0.0577, *P* = 4.93 × 10^–15^), then between hay fever and eczema (*r*_*g*_ = 0.3297, *SE* = 0.0714, *P* = 3.85 × 10^–6^) (Table 1). In summary, significant genetic correlations are observed between any pair of the three diseases. Additionally, estimates of SNP heritability (*h*^2^) on the liability scale (assuming 15% disease prevalence) is 11.85% (*SE* = 1.15%) for asthma, 4.65% (*SE* = 0.41%) for hay fever and 2.36% (*SE* = 0.53%) for eczema. Furthermore, the LD score intercepts for asthma, hay fever and eczema are 1.043 (*SE* = 0.0143), 1.0195 (*SE* = 0.0102) and 1.0085 (*SE* = 0.0105), respectively, indicating most of the inflation is due to polygenic effect rather than population structure or sample overlap (An et al., 2019).

**TABLE 1:** Genetic correlation between asthma, hay fever, and eczema.

**Diseases^1^**	**Asthma**	**Hay fever**	**Eczema**
Asthma	1	0.665 (0.0457)	0.4519 (0.0577)
Hay fever	5.256 × 10^–48^	1	0.3297 (0.0714)
Eczema	4.930 × 10^–15^	3.848 × 10^–6^	1

*^1^Element in upper off-diagonal is the genetic correlation *r*_*g*_ (standard deviation SE), element in lower off-diagonal is the corresponding genetic correlation *P*-value.*

### Pleiotropic Genes Identified by Multi-Trait Association Study

We performed metaCCA multi-trait association study to identify pleiotropic genes that are associated jointly with asthma, hay fever and eczema. There were 66 pleiotropic genes that reached the significant threshold (*P*_*metaCCA*_ < 7.6 × 10^–6^) after the Bonferroni correction of the LD pruned 6575 genes, the canonical correlations of which ranged from 0.0077 to 0.0302. The results for the metaCCA gene-based test are shown in Supplementary Data 1.

### Genes Identified by Genome-Wide and Transcriptome-Wide Studies

We conducted MAGMA genome-wide gene-based analysis to identify genes associated with asthma, hay fever and eczema, respectively. 287, 80, and 57 significant genes (*P*_*MAGMA*_ < 2.57 × 10^–6^) were identified after Bonferroni correction of the total 19427 genes (Supplementary Data 2). Moreover, we carried out MetaXcan transcriptome-wide gene-based analysis, and detected 204, 48, and 53 genes that were above the significance level (*P*_*MetaXcan*_ < 1.84 × 10^–6^) determined by 27314 genes in 10 relevant tissues (Supplementary Data 3–5).

Noticing that some overlapping genes exist for the same gene-based test, we took the results in MAGMA as an example, there are 65 overlapping genes between asthma and hay fever, 36 between asthma and eczema, 19 between hay fever and eczema, and 17 among the three diseases. Similarly, some genes detected by both MAGMA and MetaXcan for the same disease, such as 94 overlapping genes are identified in asthma. We combined the genes identified by MAGMA and/or MetaXcan, and obtained 397, 109, and 91 significant genes for asthma, hay fever and eczema, respectively. The numbers of genes identified by the two approaches are shown in Table 2.

**TABLE 2:** Number of genes identified by MAGMA and MetaXcan.

**Methods**	**Asthma**	**Hay fever**	**Eczema**	**Asthma and Hay fever**	**Asthma and Eczema**	**Hay fever and Eczema**	**Asthma and Hay fever and Eczema**
							
MAGMA	287	80	57	65	36	19	17
MetaXcan	204	48	53	37	33	5	4
Combined^1^	397	109	91	94	59	24	23

*^1^Number of genes identified by MAGMA and/or MetaXcan.*

### Shared Risk Genes for Asthma, Hay Fever, and Eczema

**FIGURE 2:**
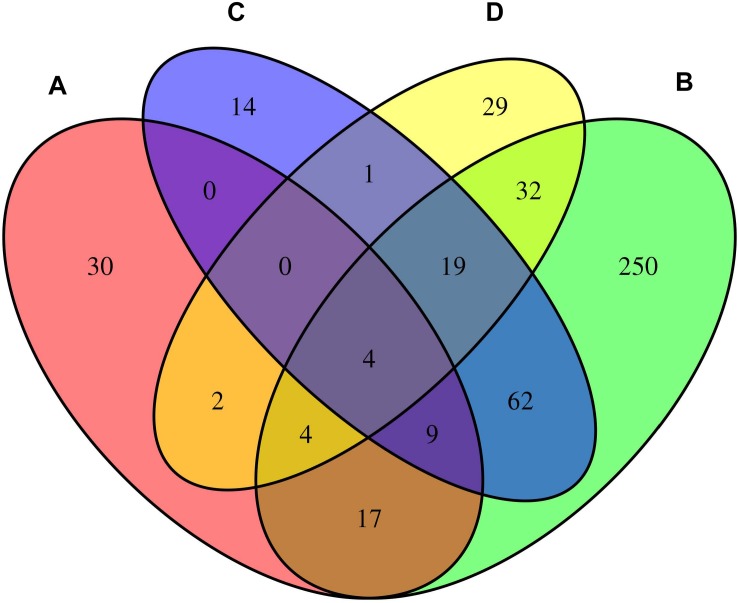
Venn diagram of the pleiotropic genes identified by metaCCA **(A)** and the combined genes identified by MAGMA and/or MetaXcan for asthma **(B)**, hay fever **(C)** and eczema **(D)**.

We considered the shared risk genes from two types. Type I includes the pleiotropic genes by metaCCA which were associated with at least one disease by MAGMA and/or MetaXcan, it is inspired by these two studies (Chen et al., 2018; Jia et al., 2019); Type II includes the pleiotropic genes associated with at least two diseases by MAGMA and/or MetaXcan. We found that type I includes 36 genes (*P*_*metaCCA*_ < 7.6 × 10^–6^, *P*_*MAGMA*_ < 2.57 × 10^–6^, and/or *P*_*MetaXcan*_ < 1.84 × 10^–6^ in at least one of asthma, hay fever and eczema), and type II contains 131 genes (*P*_*MAGMA*_ < 2.57 × 10^–6^ and/or *P*_*MetaXcan*_ < 1.84 × 10^–6^ in at least two of asthma, hay fever and eczema). After removing the repetitions in these two types, 150 shared risk genes were obtained (Supplementary Data 6). Here we only showed the details of the 17 overlapping genes in type I and II in Table 3. A Venn diagram (Figure 2) shows the pleiotropic genes identified by metaCCA and the combined genes identified by MAGMA and/or MetaXcan for asthma, hay fever and eczema. We can see that four overlap genes can not only be detected by metaCCA but also associated with all of the three diseases by MAGMA and/or MetaXcan analyses.

**TABLE 3:** Details of overlapping genes in Type I and II of shared risk genes.

**Genes^1^**	***P*_*metaCCA*_**	**Asthma**		**Hay fever**		**Eczema**		**Literature PMID**
		***P*_*MAGMA*_**	***P*_*MetaXcan*_**	***P*_*MAGMA*_**	***P*_*MetaXcan*_**	***P*_*MAGMA*_**	***P*_*MetaXcan*_**	
TNXB^†^	7.12e-29	3.51e-35		1.39e-10		1.20e-09		23886662
C6orf10‡	1.60e-18	1.59e-22		1.01e-12		9.84e-10		21804548,
								23042114
CLEC16A*	8.26e-16	4.24e-22		3.51e-10		5.92e-11		31036433,
								30013184,
								26482879
C2*	1.84e-06	1.31e-14	3.51e-21	1.08e-13		5.06e-08	1.45e-08	29551627,
								25085501,
								26542096
WDR36*	1.95e-26	1.61e-24	5.68e-14	2.58e-14	2.52e-08			30929738,
								24388013,
								30595370
PSORS1C2	3.54e-15	3.77e-13		6.80e-07				
HLA-DMB	7.72e-14		6.67e-14	3.34e-07				
BTNL2^†^	1.14e-12	1.03e-59		5.08e-09				29273806
BAG6	5.69e-11	9.44e-19	6.64e-15	7.05e-10	6.03e-18			
SLC25A46*	2.79e-09	1.35e-09		9.52e-09				31036433,
								22036096,
								30595370
CAMK4‡	2.31e-08	5.12e-11		1.28e-08				29785011,
								30013184
MUC22	8.56e-07	1.56e-13		2.01e-11				
PLCL1‡	6.08e-06	2.23e-06		9.73e-12				30013184,
								30595370
RNF5	4.31e-17	1.75e-12	3.39e-13			2.06e-10	5.84e-11	
KIF3A‡	7.05e-16	5.35e-13	6.57e-14			7.51e-08		31036433,
								26542096
DDAH2	1.78e-07		1.43e-08				3.10e-08	
RAD50*	4.05e-06	6.20e-29	9.21e-31			6.36e-07		30929738,
								30013184,
								26482879

^1^Symbol, ‡, and * behind the genes represents 1, 2, and 3 associated diseases (asthma, hay fever, eczema) reported in GWAS-catalog, respectively. PMID, PubMed unique identifier. The blank cells are non-significant *p*-values or no supporting literature.

### GWAS-Catalog Analysis, Enrichment Analysis and PPI Network Analysis

To see whether the 150 shared risk genes have been reported previously, GWAS-catalog analysis was carried out for each gene. We found 23 genes have been reported to be associated with all of the three diseases, 31 genes have been reported to be associated with two diseases, and 36 genes have been reported to be associated with one disease. Furthermore, 60 genes have never been reported, suggesting that these are novel ones. Gene names involved in these four different classes are listed in Table 4, their corresponding PubMed IDs of supporting literatures are shown in Supplementary Data 7. Among the 90 genes which have been reported as associated with diseases before, 85, 31, and 51 of them have been reported as associated with asthma, hay fever and eczema (Supplementary Data 7), respectively. Some genes are only detected by metaCCA. CGN has been reported associated with asthma, but it was not detected by MAGMA and/or MetaXcan for asthma data; RAD50 has been reported as associated with hay fever, but it was not detected by MAGMA and/or MetaXcan for hay fever data; eight genes (AHI1, IL2, MICB, NDFIP1, PLCL1, PRKCQ, SLC25A46, and WDR36) have been reported as associated with eczema, but they were not detected by MAGMA and/or MetaXcan for eczema data (Supplementary Data 6, 7). Similarly, there are also some reported genes that can only be detected by MAGMA and/or MetaXcan. 67 of the reported genes which are associated with asthma can only be successfully identified by MAGMA and/or MetaXcan, but not by metaCCA. For hay fever and eczema, gene numbers of this class are 22 and 15 (Supplementary Data 7), respectively. In addition, there are 5 genes (C2, CLEC16A, RAD50, SLC25A46, and WDR36) have been reported to be associated with all of the three diseases for the 66 pleiotropic genes by metaCCA (Supplementary Data 1). For the 424 genes (287 for asthma, 80 for hay fever, 57 for eczema) detected by MAGMA, there are 141, 23, and 24 that have been reported associated with asthma, hay fever and eczema in the GWAS-catalog (Supplementary Data 2), respectively.

**TABLE 4:** List of 150 shared risk genes divided into four categories.

**Related diseases^1^**	**Gene names**
3	BACH2, C11orf30, C2, CLEC16A, GSDMA, HLA-B, HLA-C, HLA-DQA1, IKZF3, IL13, IL18R1, IL1RL1, IL2, IL2RA, IL7R, LPP, RAD50, SLC25A46, SMAD3, TLR1, TNF, TSLP, WDR36
2	AAGAB, ADAD1, C6orf10, CAMK4, CD247, D2HGDH, ERBB3, FLG, GSDMB, HLA-DQB1, HLA-DRB1, IL18RAP, IL1R1, IL33, KIAA1109, KIF3A, MICA, MICB, NDFIP1, PBX2, PLCL1, PRKCQ, PRR5L, RORC, RPS26, RTEL1, SMARCE1, STAT6, TLR10, TMEM232, ZBTB46
1	AHI1, BRD2, BTNL2, C4A, CGN, FAM114A1, GAL3ST2, GLDC, GPSM3, HLA-DPA1, HLA-DQA2, HLA-DQB2, HLA-DRA, HLA-DRB5, HLA-DRB6, HLA-DRB9, IKZF4, IL21R, ITPR3, LCE3D, MRVI1, NOTCH4, ORMDL3, PSORS1C1, S100A1, SLC22A4, SLC22A5, SLC9A2, SLC9A4, SPRR2D, SUOX, TAP2, TLR6, TNXB, TRIM26, ZGPAT
0	AGER, AGPAT1, AIF1, ARNT, ATF6B, BAG6, BAK1, C4B, C6orf25, C6orf47, C6orf48, CCHCR1, CFB, CXXC11, CYP21A2, DDAH2, DIS3L, DOCK3, DPP4, DXO, EGFL8, EHMT2, FKBPL, GNL1, HCG27, HCG4B, HLA-DMB, HSPA1B, HSPA1L, HSPA4, KPRP, LEMD2, LINGO4, LOC101929163, LST1, MRPL9, MSH5, MUC21, MUC22, NELFE, PGLYRP4, PPT2, PRRC2A, PRRT1, PRUNE, PSMD4, PSORS1C2, RNF5, S100A2, SAPCD1, SEMA6C, SKIV2L, SLC44A4, STK19, TAP1, TCF19, TNXA, VWA7, ZBTB12, ZKSCAN3

*^1^The digit in the first column means the number of associated diseases (asthma, hay fever, eczema) reported in GWAS-catalog.*

**FIGURE 3:**
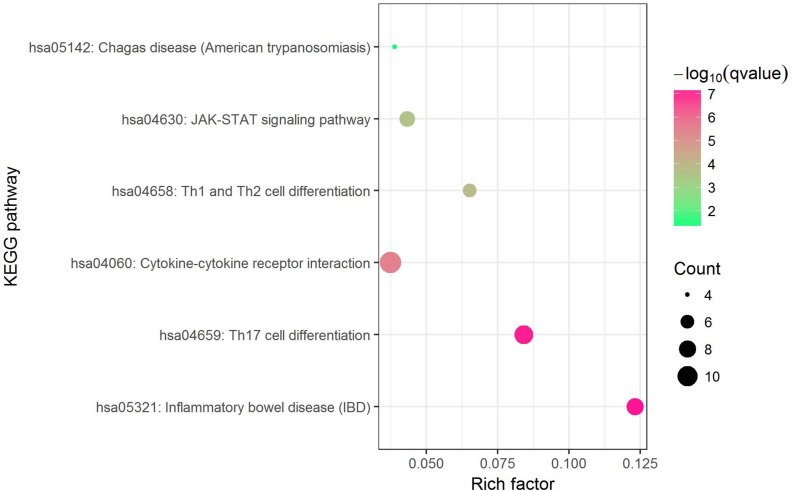
Bubble chart of enrichment analysis of shared risk genes (excluding those in MHC region).

Before conducting enrichment analysis, we excluded the genes in the major histocompatibility complex (MHC) region (Zhu et al., 2019). On the one hand, a majority of genes in MHC region are related to immune response which may bring false positives (Pividori et al., 2019); on the other hand, for asthma and allergy diseases, MHC region was reported as containing some of the strongest association signals such as HLA-DQB and HLA-B (Waage et al., 2018). We expected to find other biological pathways besides immunity. KEGG pathway enrichment analysis by Enrichr web server (http://amp.pharm.mssm.edu/Enrichr) shows that 6 biological pathways were significantly enriched (Supplementary Data 8). They are inflammatory bowel disease (IBD) (hsa05321), Th17 cell differentiation (hsa04659), cytokine–cytokine receptor interaction (hsa04060), Th1 and Th2 cell differentiation (hsa04658), JAK-STAT signaling pathway (hsa04630) and chagas disease (American trypanosomiasis) (hsa05142). The most strongly enriched one is IBD pathway (hsa05321) including 8 enriched genes (IL18RAP, SMAD3, IL13, RORC, IL21R, STAT6, IL2, IL18R1). A bubble chart shows the result of KEGG pathway analysis (Figure 3).

**FIGURE 4:**
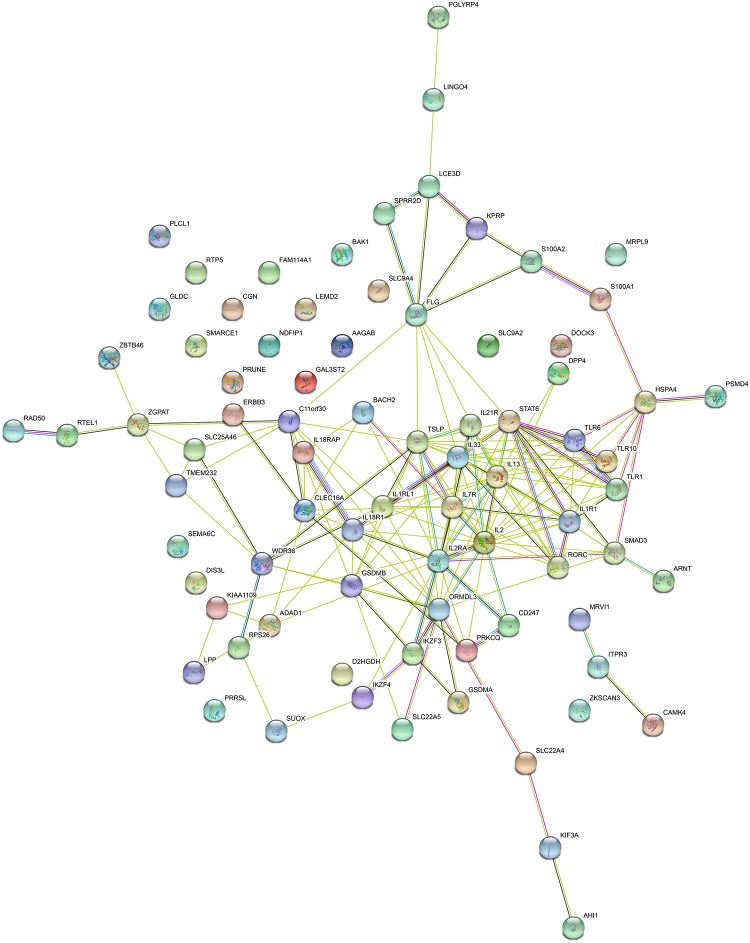
PPI network for shared risk genes (excluding those in MHC region).

To understand the interactions between shared risk genes (excluding those in MHC region), we conducted PPI network analysis using STRING tool. There are in total 168 pairs of interaction in PPI network (Supplementary Data 9), all the interacting genes have combined scores of no less than 0.4, in which 9 pairs of genes (IL2RA-IL2, IL33-IL1RL1, TSLP-IL7R, IL18R1-IL18RAP, IL13-STAT6, IKZF3-IL2, CD247-IL2, LCE3D-SPRR2D, TLR6-TLR1) with scores ≥ 0.95. The 10 hub genes (degree ≥ 10) that interact extensively with other genes in PPI network are IL2, IL13, TSLP, IL2RA, IL33, STAT6, ORMDL3, IL1R1, IL1RL1 and IL7R. The PPI network for shared risk genes are shown in Figure 4.

## Discussion

Two-thirds of our identified shared risk genes were reported to associate with at least one of the three diseases, asthma, hay fever and eczema. Results obtained by Enrichment analysis are mostly consistent with the findings in previous researches. For example, we found substantial shared genes in the HLA region, which was highlighted by their prominent role in immune response (Pividori et al., 2019), and immune response is one of the major factors influencing asthma, hay fever and eczema (Ferreira et al., 2017; Zhu et al., 2018). Additionally, IBD pathway (hsa05321) is the most strongly enriched pathway in our study, which was demonstrated to share susceptibility genes with allergic disease (Kreiner et al., 2017). Moreover, there are also some T cell (including TH17, TH1, TH2) related pathways enriched, involving Th17 cell differentiation (hsa04659), Th1 and Th2 cell differentiation (hsa04658). This conclusion supports that of a previous study which widely documented contribution of these T cell subsets to allergic responses (Farh et al., 2015).

We found four genes (C2, CLEC16A, C6orf10, TNXB) which have statistical significance in metaCCA, MAGMA and MetaXcan association studies for the three diseases. C2 and CLEC16A have been reported to associate with all the three diseases (Waage et al., 2018; Zhu et al., 2018; Kichaev et al., 2019). Although TNXB has only been reported to associate with eczema (Baurecht et al., 2015), it may be very important for asthma and hay fever. Among the 17 overlapping genes from types I and II of shared risk genes, six genes (PSORS1C2, HLA-DMB, BAG6, MUC22, RNF5, DDAH2) have never been reported before. Furthermore, cytokine-cytokine receptor interaction (hsa04060), JAK-STAT signaling pathway (hsa04630) and chagas disease (American trypanosomiasis) (hsa05142) also enriched in our study. These findings may be helpful in pathological diagnosis studies.

From the single-trait GWAS results of asthma, hay fever and eczema, only one independent loci (rs61893460) is found to associate with these three diseases. rs61893460 locates in C11orf30-LRRC32 region on chromosome 11 and was reported associated with total serum IgE levels (Li et al., 2012). IgE is released from the immune system and travels to local organs or tissues to type 2 cytokines, which can further cause asthma, hay fever and eczema (Ferreira et al., 2017). However, metaCCA multi-trait analysis identifies 66 pleiotropic genes, which implies stronger statistical power. We did not regard all of the 66 pleiotropic genes as shared risk genes, but refined them under a restraint, that is, they must be associated with at least one of the three diseases by MAGMA/MetaXcan. This idea derives from the two studies (Chen et al., 2018; Jia et al., 2019).

Using multi-trait analysis, we only identified five genes which have been reported associated with the three diseases, while 23 reported genes are detected by integrating multi-trait and multiomic methods. In addition, among the 90 genes which have been reported, some cannot be detected by a single method. Take gene RAD50 for example, it was reported to be associated with the three diseases in GWAS-catalog and can be identified by multi-trait method (metaCCA), but it cannot be detected by multiomic methods (MAGMA and/or MetaXcan) for hay fever disease. RAD50 promotes the development of asthma by inducing inflammatory factors secreted by Th2 cell (Li et al., 2010), and it was found to be associated with hay fever (Waage et al., 2018). These results imply the benefits of integration.

Note that 73 of 136 independent risk variants are novel in Ferreira et al. (2017), 41 of 141 loci are novel in Johansson et al. (2019), and 60 of 150 shared risk genes are novel in our study. Besides the different phenotypic definitions which we have explained in the Introduction section, the determining of novel status is also different. The novel variants not only included those risk loci that never reported to associate with any of the three diseases in GWAS-catalog, but also contained the variants that had LD statistic *r*^2^ < 0.05 with all reported variants (Ferreira et al., 2017). Moreover, the novel loci were composed of variants if the locus was distanced >1 Mb from any of the previously reported loci for any of the three diseases in GWAS-catalog, PubMed or bioRxiv, as well as those variants if *r*^2^ < 0.05 between the identified variant and previously reported variants (Johansson et al., 2019). Both of the definitions of “novel” in these two studies are broader than ours. In addition, we investigated genetic overlap on gene level rather than genetic variant level.

Compared with the previous studies, our work has some achievements. First, we confirmed strong genetic correlations between the three diseases. Second, we considered the pleiotropic effects via multi-trait association analysis, which yields a statistical power advantage compared to single-trait modeling strategies. Third, we identified more shared risk genes from multi-omic (genome-wide and transcriptome-wide) perspective.

### Limitations

First, our results cannot be used to represent the worldwide population or children, because the samples are of European-ancestry individuals aged between 40 and 69 years old from UK Biobank. Second, association studies results in our work mean potential shared risk genes, they do not represent the causative genes. Mendelian randomization analysis can be used to reveal the causality (Verbanck et al., 2018), and fine mapping is helpful in detecting the pathogenic variants and genes (Marenholz et al., 2013; Farh et al., 2015). Third, the functions of novel shared risk genes are still unknown. There is a long way to go in understanding the gene functions and their roles in disease pathophysiology. Further studies should also highlight and explore the biological interpretation and try to translate the findings to clinical research or practice.

## Conclusion

We confirmed strong genetic correlations between asthma, hay fever and eczema. Three different association studies are integrated to identify the shared risk genes between these three diseases. One is metaCCA multi-trait association analysis considering the joint effect, another two are MAGMA and MetaXcan gene-based tests using genome-wide and transcriptome-wide data referring to 1000 Genomes and GTEx project, respectively. We identified 150 shared risk genes, in which 60 are novel. Functional enrichment analysis reveals that the shared risk genes are enriched in inflammatory bowel disease (IBD), T cells differentiation and other related biological pathways. Our work may provide help on treatment of asthma, hay fever and eczema in clinical application.

## Data Availability Statement

The GWAS result datasets analyzed for this study can be found in the GeneALTAS http://geneatlas.roslin.ed.ac.uk/.

## Author Contributions

HG conceived the project, performed the data analysis, and wrote the manuscript. JA participated in guidance and discussion. ZY contributed to guidance and supervised the project. All authors read and approved the final manuscript.

## Conflict of Interest

The authors declare that the research was conducted in the absence of any commercial or financial relationships that could be construed as a potential conflict of interest.
